# Chemotherapy-refractory high-grade serous ovarian carcinoma: single-cell findings, TCGA-OV transcriptomic context, and a single-case MRI–transcriptome illustration

**DOI:** 10.3389/fgene.2026.1943981

**Published:** 2026-10-19

**Authors:** Litian Chen, Pingping Fu, Sunjie Wu, Ben Chen, Xin Wen

**Affiliations:** 1 Radiology Imaging Center, Nanbai Xiang Hospital Complex, The First Affiliated Hospital of Wenzhou Medical University, Wenzhou, Zhejiang, China; 2 Gynecology Department, The First Affiliated Hospital of Wenzhou Medical University, Wenzhou, Zhejiang, China

**Keywords:** chemotherapy refractoriness, high-grade serous ovarian carcinoma, hypoxia, magnetic resonance imaging, radiogenomics, single-cell RNA sequencing, The Cancer Genome Atlas Ovarian Cancer, type-I interferon

## Abstract

**Background:**

Primary chemotherapy refractoriness remains a clinical challenge in high-grade serous ovarian carcinoma (HGSC) and may reflect pre-existing tumor-cell states and microenvironmental heterogeneity. We examined public single-cell and bulk transcriptomic resources, a barcode-matched MRI case, and an *in vitro* cisplatin-perturbation RT-qPCR experiment.

**Methods:**

We reanalyzed GSE300897, comprising 19,156 annotated cells from nine treatment-naïve HGSC biopsy libraries (four chemotherapy-refractory and five chemotherapy-sensitive), with response summaries retained at the library/patient level. The Cancer Genome Atlas Ovarian Cancer (TCGA-OV) provided 421 unique primary tumors for cohort-scale transcriptomic and overall survival context. One TCGA-OV case (TCGA-24-1423) linked a multiparametric MRI examination to a tumor RNA-seq barcode. OVCAR-3 and Caov-3 cells were exposed to cisplatin (5 μM, 24 h), and IFIT1, MX1, SNAI1, and HIF1A expression was measured by RT-qPCR.

**Results:**

Across the nine GSE300897 libraries, no cancer-cell program passed Benjamini–Hochberg (BH) *q* < 0.05. Refractory libraries showed a lower type-I interferon (IFN-I) score (−0.15 log1p(CP10k)) and epithelial-to-mesenchymal transition (EMT) balance (−0.16), with a small increase in hypoxia (+0.05). In TCGA-OV, none of the six displayed program scores reached BH *q* < 0.05 in age- and stage-adjusted Cox models; IFN-I tertiles separated nominally by Kaplan–Meier analysis (log-rank *p* = 0.029), whereas continuous IFN-I was not significant after adjustment (HR = 0.96 per SD, BH *q* = 0.927). In TCGA-24-1423, the paired transcriptome showed an IFN-I score at the 86.8th TCGA-OV percentile, with IFIT1 and MX1 at the 87.5th and 93.7th percentiles. RT-qPCR showed lower IFIT1/MX1 and higher SNAI1/HIF1A after acute cisplatin exposure in both cell lines (all Holm-adjusted *p* < 0.05).

**Conclusion:**

These findings support a conservative, hypothesis-generating interpretation of chemotherapy response-associated transcriptional heterogeneity in HGSC. Reduced IFN-I and altered cell-state programs were directional features of the small refractory single-cell cohort, whereas TCGA-OV provided context rather than independent response validation. The MRI-linked case demonstrates imaging–transcriptome pairing but does not establish an imaging biomarker. The RT-qPCR experiment demonstrates cisplatin-responsive changes in selected genes but models acute drug perturbation rather than the pretreatment clinical refractory state.

## 1 Introduction

High-grade serous ovarian carcinoma (HGSC) accounts for a substantial proportion of ovarian cancer mortality and is commonly treated with cytoreductive surgery and platinum-based chemotherapy (Sung et al., 2021; Lheureux et al., 2019; Bowtell et al., 2015). Although many patients initially respond, a clinically important subset has primary refractory disease, and acquired platinum resistance is also frequent, underscoring the need to define pretreatment biological states associated with poor chemotherapy response (Patch et al., 2015; Afenteva et al., 2025).

HGSC is molecularly and cellularly heterogeneous (Kandalaft et al., 2022). Large-scale genomic and proteogenomic profiling has established extensive copy-number alteration, recurrent pathway-level diversity, and transcriptional subtypes (Cancer Genome Atlas Research Network, 2011; Tothill et al., 2008; Konecny et al., 2014; Chen et al., 2018; Zhang et al., 2016). Single-cell studies have further resolved malignant, immune, stromal, and vascular compartments that can contribute differently to clinical behavior and treatment response (Izar et al., 2020; Olbrecht et al., 2021; Xu et al., 2022; Qian et al., 2020). Epithelial–mesenchymal plasticity, hypoxia, interferon signaling, immune states, and DNA-damage/drug-handling programs are therefore useful descriptive axes for organizing this heterogeneity, but transcriptomic program scores should not be interpreted as direct evidence of a causal mechanism (Nieto et al., 2016).

The public GSE300897 resource originates from a multi-omics study of treatment-naïve HGSC that reported attenuated type-I interferon (IFN-I) activity and increased hypoxia-associated signaling in chemo-refractory disease (Afenteva et al., 2025). Type-I interferon signaling can modulate tumor-cell responses to chemotherapy, while hypoxic adaptation is a recognized contributor to chemoresistant phenotypes across solid tumors (Sistigu et al., 2014; Belisario et al., 2020). In the present work, we use the processed single-cell data as a secondary reanalysis and retain the patient/library as the unit of response comparison. This distinction is important: our analysis is intended to test whether directional program patterns remain visible under a transparent library-level scoring framework, not to reproduce or extend the original study’s functional experiments.

To place the single-cell findings in a broader disease context, we additionally analyze primary-tumor transcriptomes from TCGA-OV and illustrate barcode-level linkage between one TCGA-OV RNA-seq sample and its publicly available multiparametric MRI examination. Prior ovarian cancer radiogenomic work shows that imaging and genomic resources can be paired to investigate phenotype–genotype relationships (Vargas et al., 2017), while multiparametric MRI can capture complementary anatomic and diffusion-sensitive information relevant to tumor heterogeneity (Just, 2014). The MRI component is deliberately limited to a single case from the TCGA-OV imaging collection (Holback et al., 2016). Accordingly, the study aims to triangulate evidence across distinct data layers rather than develop a clinical imaging classifier or treatment-selection biomarker. A separate *in vitro* RT-qPCR experiment in OVCAR-3 and Caov-3 cells characterized acute cisplatin-responsive expression of selected IFN-I, EMT-related, and hypoxia-associated genes; this perturbation assay was not designed as a direct validation of the pretreatment refractory-versus-sensitive state.

## 2 Methods

### 2.1 Study scope and data sources

This study integrated three public data components and one *in vitro* RT-qPCR experiment. GSE300897 supplied processed single-cell profiles from nine treatment-naïve HGSC biopsy libraries (four chemotherapy-refractory and five chemotherapy-sensitive). TCGA-OV provided bulk RNA-seq and clinical context for primary ovarian serous carcinoma. One TCGA-OV case (TCGA-24-1423) linked a multiparametric MRI examination to a tumor RNA-seq barcode. The public data components were analyzed at their native units: library/patient summaries for response comparisons, one primary tumor per TCGA patient, and barcode-level pairing for the MRI case. The cell line experiment was analyzed separately as an acute cisplatin-perturbation assay.

### 2.2 Single-cell processing and analysis unit

The GSE300897 annotation contained 19,156 cells from nine libraries/patients, and the corresponding GEO unique molecular identifier (UMI) matrix contained 58,037 genes and 19,156 cell columns. For plotted genes, raw UMI counts were normalized in each cell by the annotation-provided total UMI count and transformed as log1p(CP10k). Cell subtype, treatment status, and anatomical location were retained as supplied. Response-oriented summaries were aggregated at the library/patient level, while cell-level projections and dot plots visualized annotation and program structure. All three omental libraries belonged to the sensitive group, so status and location were displayed together when interpreting response-associated patterns. General single-cell preprocessing and visualization principles were interpreted in the context of established best-practice guidance (Luecken and Theis, 2019).

### 2.3 Gene programs

Predeclared canonical gene panels summarized type-I interferon signaling, hypoxia, mesenchymal and epithelial EMT components, cytotoxicity, immune-inhibitory genes, macrophage-like M1/M2 programs, extracellular matrix genes, and a DNA-damage/drug-handling expression program. In the single-cell analysis, a program score was the mean log1p(CP10k) expression across the available panel genes. EMT balance was defined as the mesenchymal score minus the epithelial score. The same predefined gene panels and averaging framework were retained for TCGA-OV, with modality-specific normalization as described below.

### 2.4 TCGA-OV bulk transcriptomics and clinical context

TCGA-OV raw counts were restricted to one primary tumor per patient (421 samples/patients); eight recurrent tumors were excluded. Counts were transformed to log2(CPM+1), and each gene was standardized across the 421 primary tumors before program scores were calculated. A total of 419 cases were evaluable for the survival analyses shown. Vital status comparisons were cross-sectional. Cox models for overall survival included age and stage IV versus stage IIIC among evaluable stage IIIC/IV cases; proportional-hazards test *p* values are displayed with the estimates. TCGA-OV supplied transcriptomic and overall survival context, while refractory-versus-sensitive comparisons were anchored to GSE300897. Benjamini–Hochberg false-discovery-rate correction (Benjamini and Hochberg, 1995) was applied separately within the displayed analysis families: across cancer-cell response-program comparisons in GSE300897 and across the six displayed TCGA Cox program tests. Cox regression and proportional-hazards diagnostics followed standard formulations (Cox, 1972; Grambsch and Therneau, 1994).

### 2.5 MRI case display

The TCGA-24-1423 examination contained 23 MR series and 723 DICOM instances from a 1.5 T abdominal-pelvic study. Representative central images from pre-contrast, post-contrast, and delayed T1 VIBE, axial T2 HASTE, diffusion-weighted imaging, and apparent diffusion coefficient (ADC) series were displayed after percentile-based windowing. These six acquisitions summarize contrast timing, fluid-sensitive soft-tissue contrast, and diffusion information in the barcode-matched case. The paired RNA-seq profile was positioned within the 421-primary-tumor TCGA-OV distribution by program and gene percentiles.

### 2.6 *In vitro* RT-qPCR experiment

NIH:OVCAR-3 (ATCC HTB-161) and Caov-3 (ATCC HTB-75) were used as ATCC-derived human ovarian cancer cell models. OVCAR-3 cells were cultured in RPMI-1640 supplemented with 20% fetal bovine serum and 0.01 mg/mL bovine insulin, whereas Caov-3 cells were cultured in DMEM supplemented with 10% fetal bovine serum; both were maintained at 37 °C and 5% CO_2_. These lines were selected as established ovarian cancer models, while acknowledging that no cell line fully recapitulates primary HGSC biology (Domcke et al., 2013; Beaufort et al., 2014). Cells at approximately 70–80% confluence were exposed to cisplatin (5 μM) or vehicle for 24 h. Total RNA was extracted using TRIzol and reverse transcribed, followed by SYBR Green RT-qPCR for IFIT1, MX1, SNAI1, and HIF1A, with GAPDH as the internal reference. Four independent cultures per condition were analyzed, with each cDNA measured in technical triplicate. Relative expression was calculated by the 2^−ΔΔCt^ method (Livak and Schmittgen, 2001), using the vehicle condition as the calibrator. Statistical testing was performed on replicate-level ΔΔC_t_ values using two-sided one-sample *t* tests against 0 with Holm adjustment across the four genes within each cell line. The RT-qPCR experiment was analyzed separately as an acute cisplatin-perturbation assay and was not combined statistically with GSE300897 or TCGA-OV.

## 3 Results

### 3.1 Dataset architecture and sample composition

The single-cell component comprised 19,156 annotated cells from nine treatment-naïve HGSC libraries. Library sizes varied across samples, with supplied cell subtype labels supporting explicit coverage inspection (Figure 1A). The library-by-cell-type heatmap identifies the samples contributing to each compartment (Figure 1B), while within-library composition is shown without pooling cells across patients (Figure 1C). Mean library-level fractions differed across response groups (Figure 1D). Supplied per-cell UMI, detected-gene, and mitochondrial metrics provide quality control (QC) context (Figure 1E). The status-by-location table shows that refractory and sensitive cells were distributed differently across peritoneum, mesentery, and omentum (Figure 1F).

**FIGURE 1:**
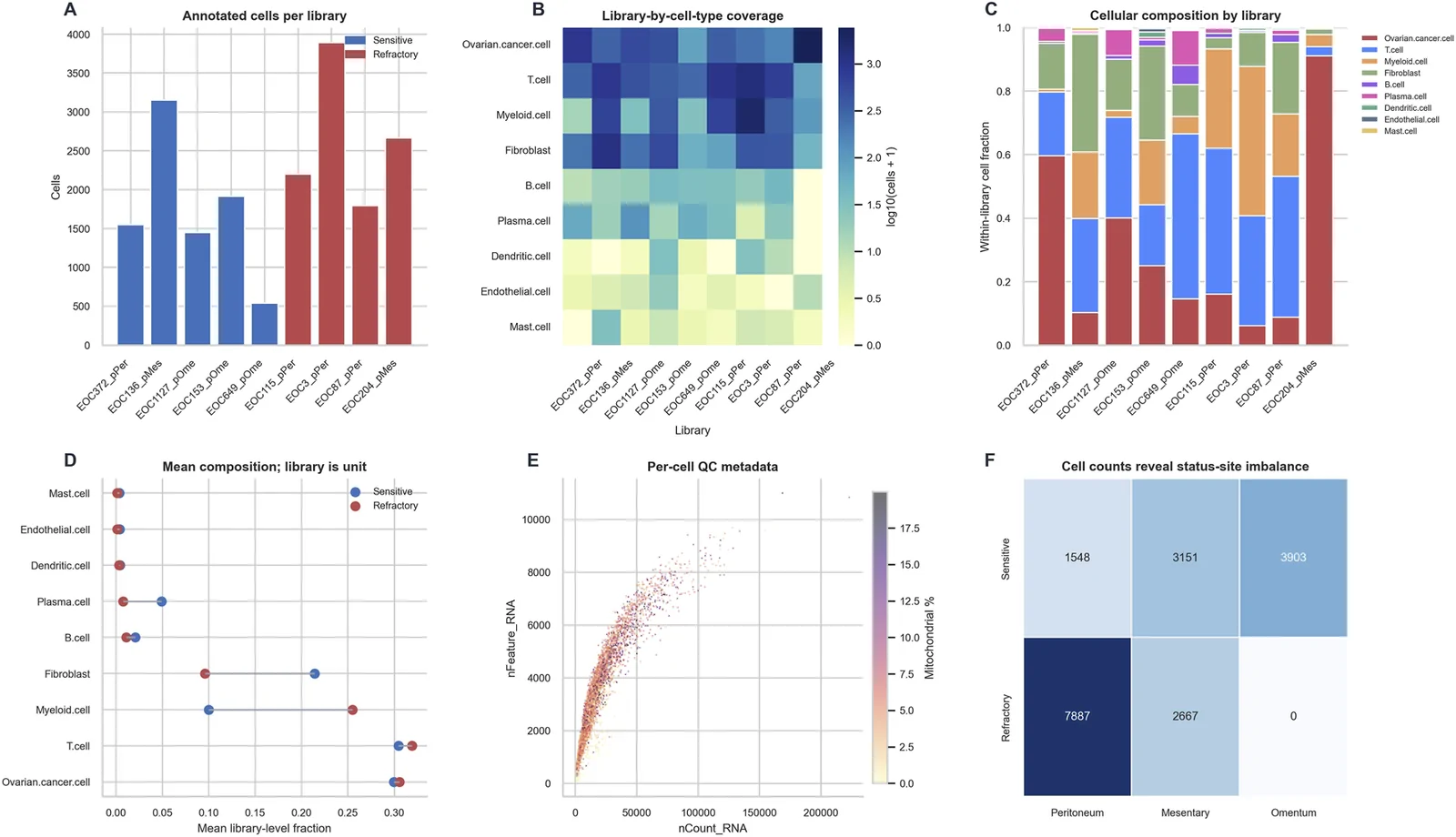
GSE300897 data architecture and library-level QC. **(A)** Annotated cell count per library. **(B)** Cell-type coverage by library; color is log10(cells+1). **(C)** Within-library composition. **(D)** Mean library-level cell-type fractions by chemotherapy status; each library contributes equally within a group. **(E)** Supplied per-cell QC metadata. **(F)** Cell-count cross-tabulation of status and anatomical location. Response and location are visibly imbalanced.

### 3.2 Selected-gene embedding visualizes cellular annotations and programs

A uniform manifold approximation and projection (UMAP) constructed from 129 pre-declared marker/program genes placed supplied annotations and expression programs in a common low-dimensional space. The projection was colored by supplied cell subtype (Figure 2A), chemotherapy status (Figure 2B), biopsy location (Figure 2C), and library (Figure 2D). IFN-I and EMT-balance overlays identify program variation across the same fixed projection (Figures 2E, F).

**FIGURE 2:**
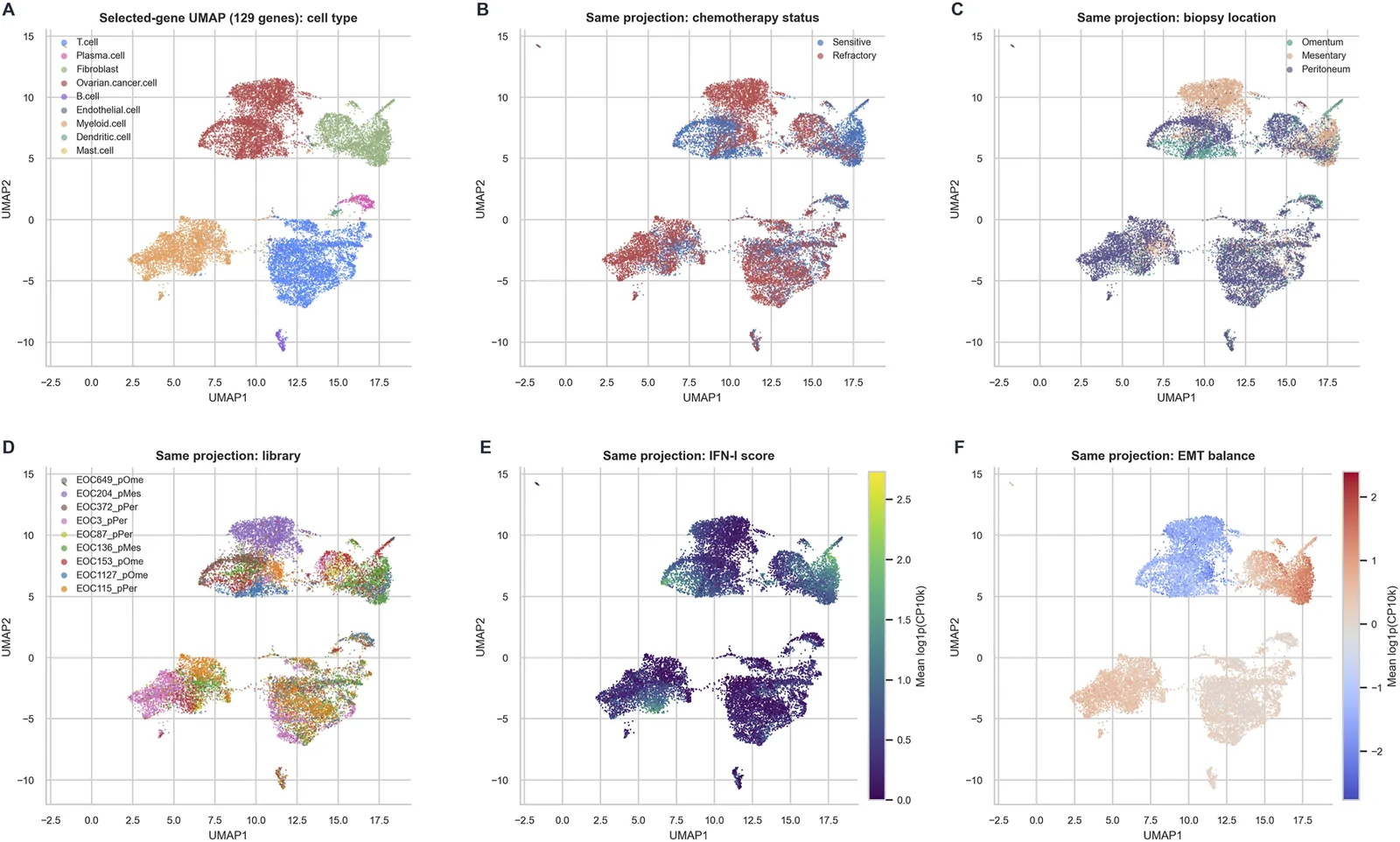
Selected-gene UMAP of GSE300897. The projection uses 129 pre-declared marker/program genes after log1p(CP10k) normalization. **(A)** Supplied cell subtype. **(B)** Chemotherapy status. **(C)** Anatomical location. **(D)** Library. **(E)** IFN-I score. **(F)** EMT balance. Library-level response patterns are summarized in Figures 4–6.

### 3.3 Cellular compartments carry distinct marker and program patterns

Marker detection and mean expression distinguish the supplied epithelial, immune, stromal, and vascular compartments (Figure 3A). Program averaging highlights compartmentalized IFN-I, hypoxia, EMT, cytotoxic, inhibitory, and extracellular matrix (ECM) signals (Figure 3B). Library-level fractions for the six most abundant compartments retain individual-library points rather than treating cells as independent replicates (Figure 3C). All cell types remain auditable by library (Figure 3D), and immune/hypoxia-associated genes distribute across multiple compartments (Figure 3E). Cancer-cell and myeloid-cell fractions are displayed jointly at the library level as a compositional relationship (Figure 3F).

**FIGURE 3:**
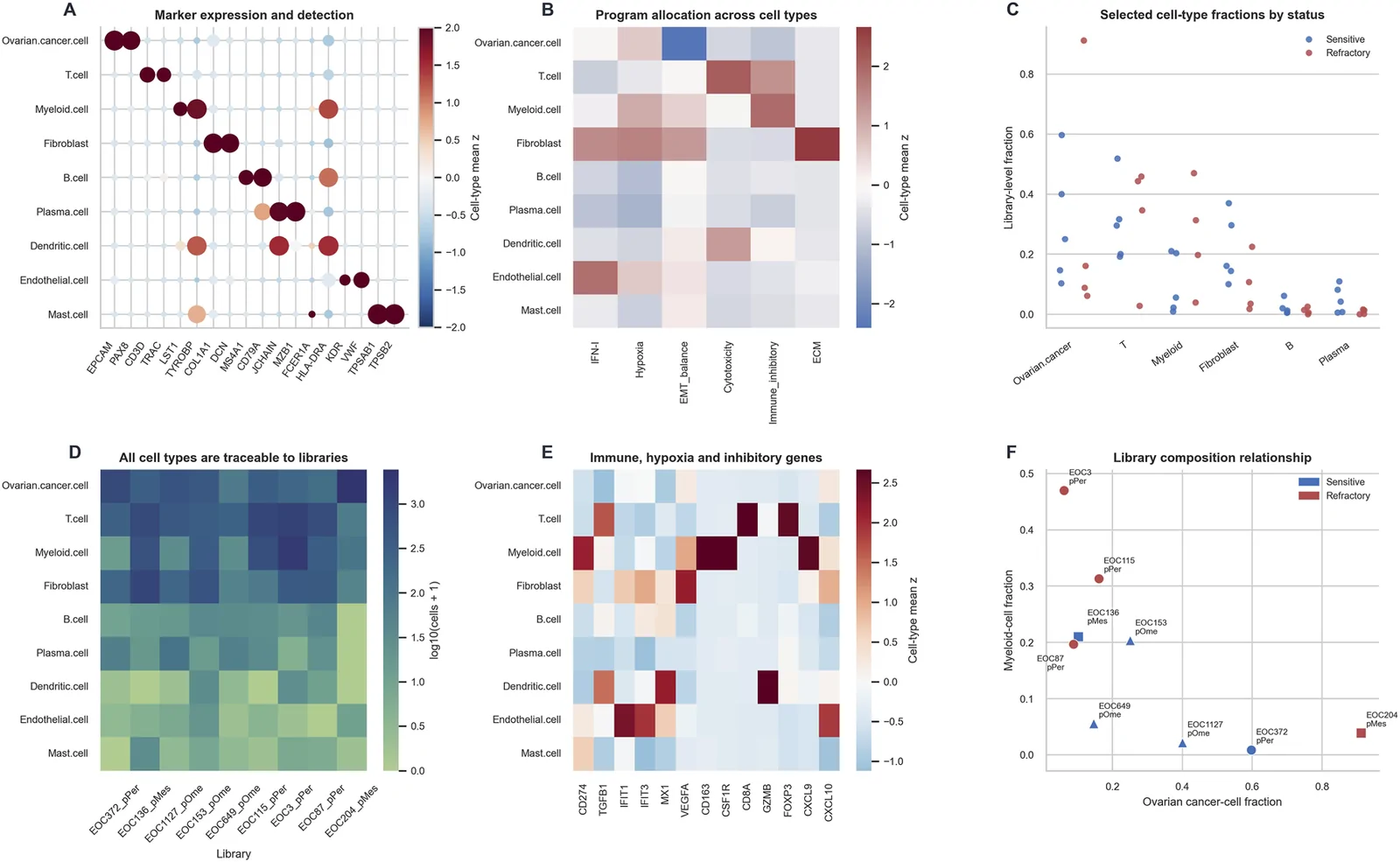
Cellular annotation and program allocation in GSE300897. **(A)** Marker dot plot: color is cell-type mean z score, and area is detection fraction. **(B)** Cell-type mean program score heatmap. **(C)** Selected compartment fractions by library/status. **(D)** Cell-type library coverage. **(E)** Immune, hypoxia, and inhibitory gene heatmap. **(F)** Cancer-cell versus myeloid-cell library fractions. Composition panels use libraries as the unit.

### 3.4 Cancer-cell programs provide exploratory response-associated patterns

The malignant epithelial compartment was represented in each library, although both absolute cell number and within-library fraction varied (Figure 4A). At the patient/library level, cancer-cell IFN-I scores differed by an average of −0.15 log1p(CP10k) units between refractory and sensitive libraries (BH *q* = 0.476; Figure 4B). Cancer-cell hypoxia scores showed an average refractory-minus-sensitive difference of 0.05 (BH *q* = 0.730; Figure 4C). EMT balance (Figure 4D) and the DNA-damage/drug-handling expression program (Figure 4E) are shown with the same library-level unit. The integrated heatmap preserves heterogeneity across all nine cancer-cell libraries (Figure 4F).

**FIGURE 4:**
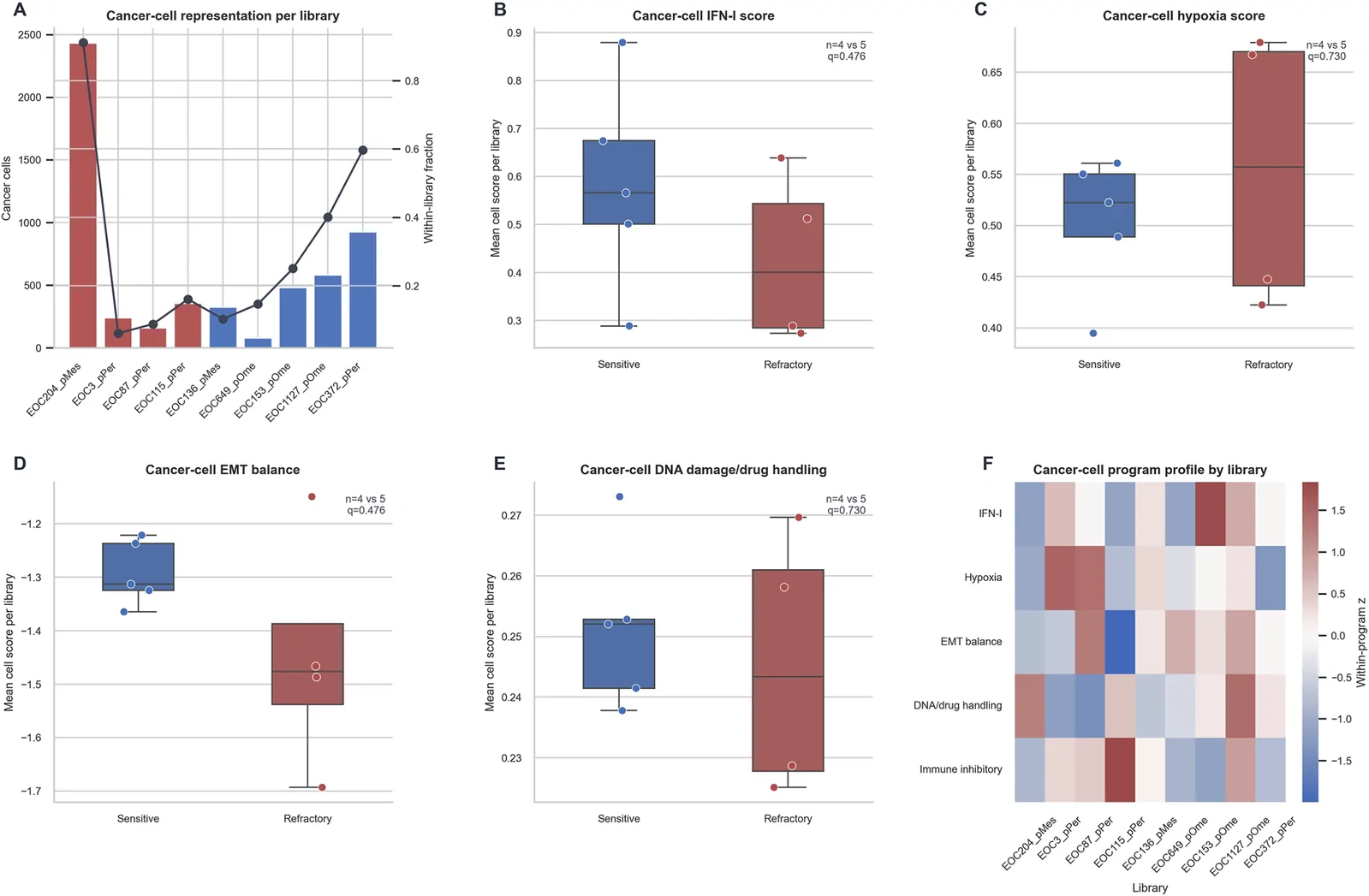
Cancer-cell response-associated patterns in GSE300897. **(A)** Cancer-cell number and within-library fraction. **(B)** IFN-I, **(C)** hypoxia, **(D)** EMT balance, and **(E)** DNA-damage/drug-handling scores, aggregated over cancer cells within each library. *q* values are BH-adjusted across the displayed programs. **(F)** Within-program z-score heatmap by library. Four refractory and five sensitive libraries are shown; the status/location structure is detailed in Figure 6D.

### 3.5 Immune and stromal programs remain compartment-specific

T-cell, myeloid-cell, and fibroblast fractions are compared as library-level quantities (Figure 5A). T-cell cytotoxicity and inhibitory scores are displayed jointly for each available library (Figure 5B), while M1-like and M2-like myeloid scores are shown separately (Figure 5C). Fibroblast ECM and hypoxia programs are likewise summarized by library (Figure 5D). The cross-compartment distribution of checkpoint, interferon, hypoxia, and immune-lineage genes is visualized by a cell-type dot plot (Figure 5E). Finally, T-cell cytotoxicity and myeloid M2-like values are plotted across matched libraries as an exploratory ecological relationship (Figure 5F).

**FIGURE 5:**
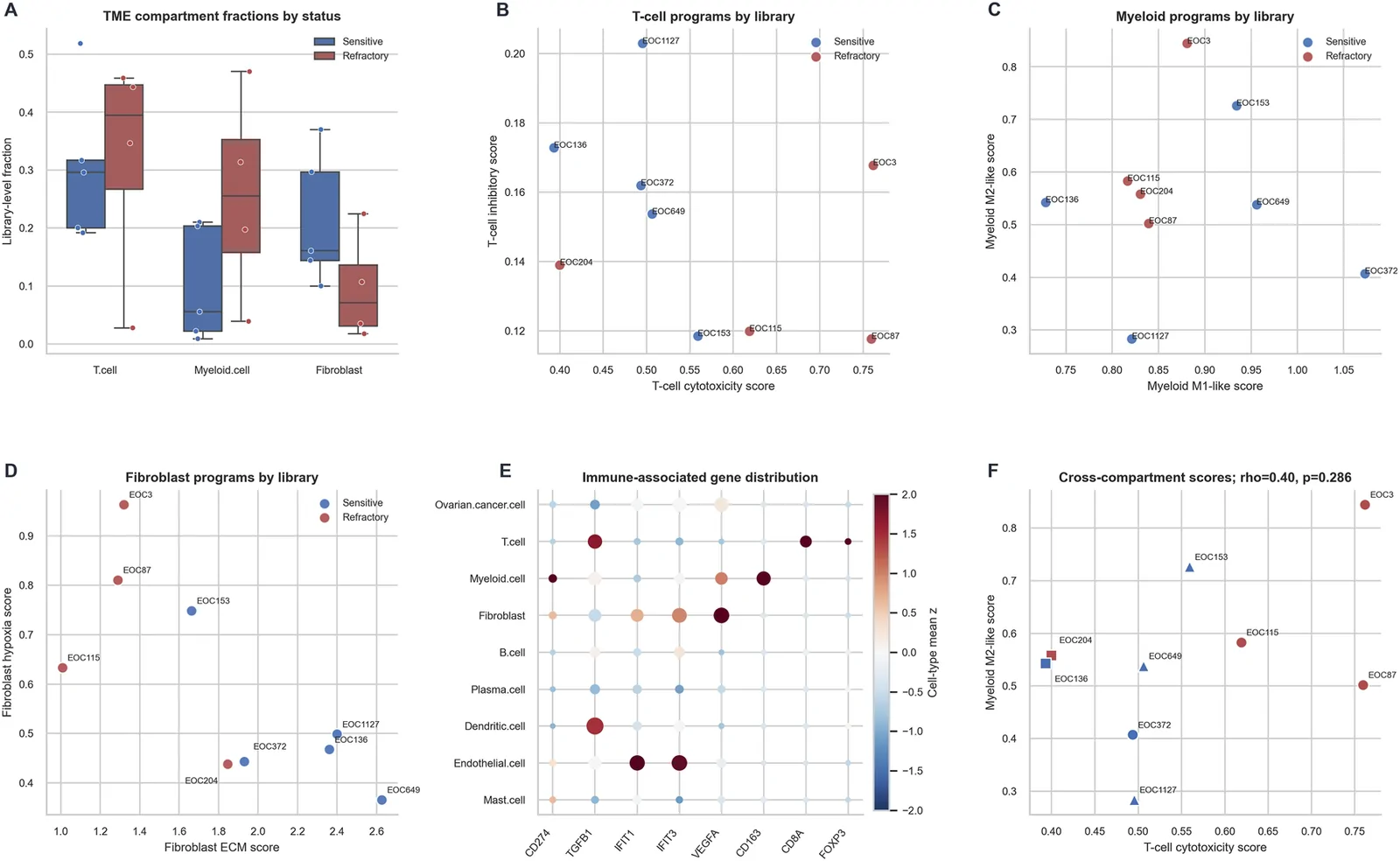
Compartment-specific tumor microenvironment descriptions. **(A)** T-cell, myeloid-cell, and fibroblast fractions by library/status. **(B)** T-cell cytotoxicity and inhibitory scores. **(C)** Myeloid M1-like and M2-like scores. **(D)** Fibroblast ECM and hypoxia scores. **(E)** Cell-type dot plot of selected immune-associated genes. **(F)** Matched-library association of T-cell cytotoxicity and myeloid M2-like score. All quantitative group displays retain the library as the analytical unit.

### 3.6 Sample-level integration connects program effects to study design

Selected-gene pseudobulk principal component analysis (PCA) treats each library as one point and shows both status and location annotations (Figure 6A). Cancer-cell program correlations are calculated across the nine libraries (Figure 6B). Refractory-minus-sensitive effect sizes and BH-adjusted *p* values are reported at the library level (Figure 6C). The binary sample-design matrix shows the non-overlap of omental location with refractory status (Figure 6D). IFN-I and hypoxia score relationships are shown at the same unit (Figure 6E), and the ordered IFN-I profile makes the response/location composition of the ranking visible (Figure 6F).

**FIGURE 6:**
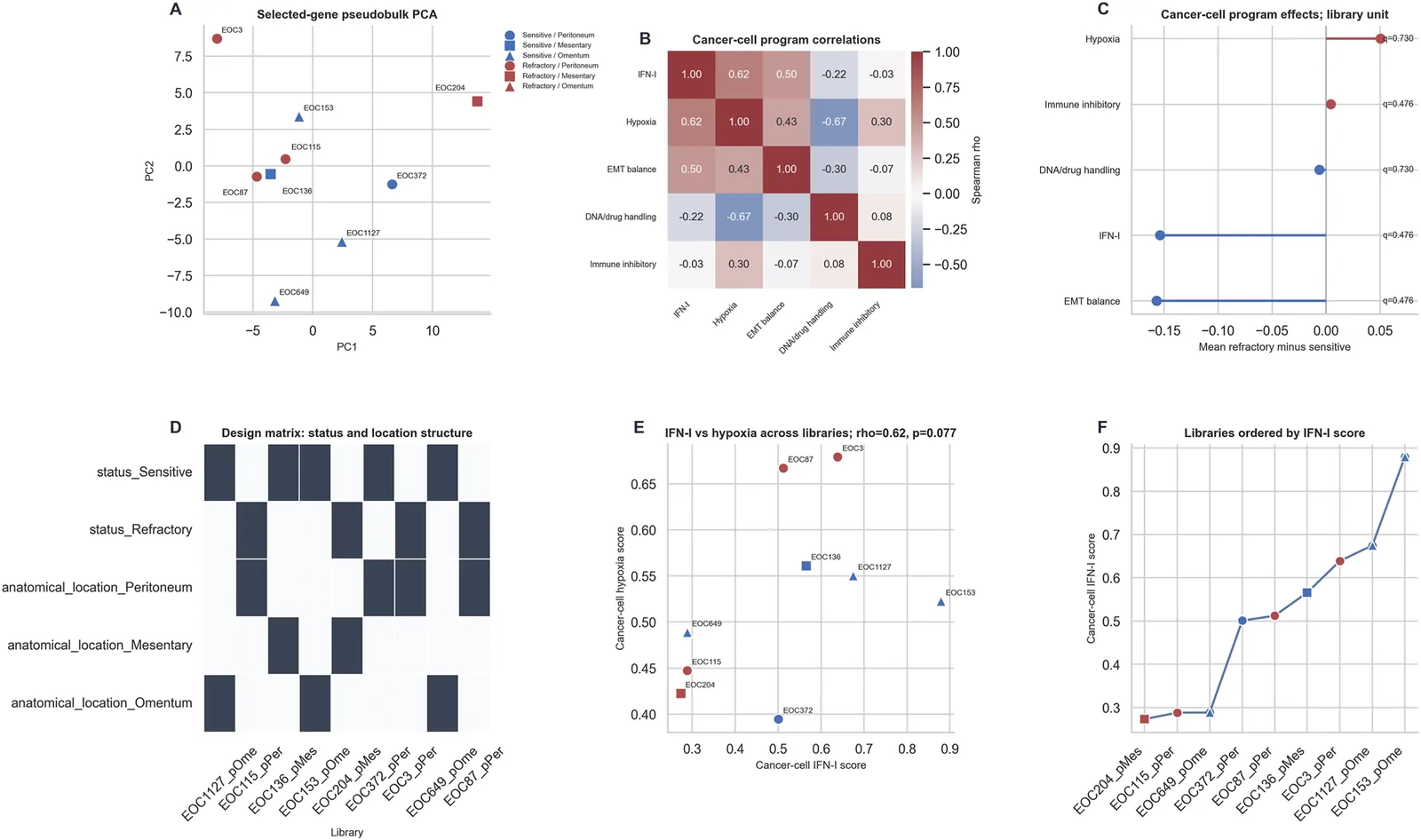
Sample-level integration of GSE300897. **(A)** PCA of selected-gene pseudobulk profiles, one point per library. **(B)** Spearman correlations of cancer-cell program scores across nine libraries. **(C)** Refractory-minus-sensitive effect sizes with BH *q* values. **(D)** Status/location design matrix. **(E)** Cancer-cell IFN-I versus hypoxia score. **(F)** Libraries ordered by cancer-cell IFN-I score. The design matrix supplies the location context for reading the effect panels.

### 3.7 TCGA-OV contextualizes program variation and overall survival

TCGA-OV contributed 421 unique primary tumors, with 419 cases evaluable for the displayed survival analysis (Figure 7A). Program distributions by vital status show broad overlap (Figure 7B), and stage-wise medians vary modestly without a consistent ordinal pattern (Figure 7C). In age- and stage-adjusted Cox models, 0 of six program scores reached BH *q* < 0.05 (Figure 7D). The IFN-I tertile Kaplan–Meier curves separated nominally (log-rank *p* = 0.029; Figure 7E), whereas continuous IFN-I was not significant after adjustment (HR = 0.96 per SD, BH *q* = 0.927). IFN-I and EMT balance were only weakly related across tumors (Spearman rho = −0.07, *p* = 0.163; Figure 7F).

**FIGURE 7:**
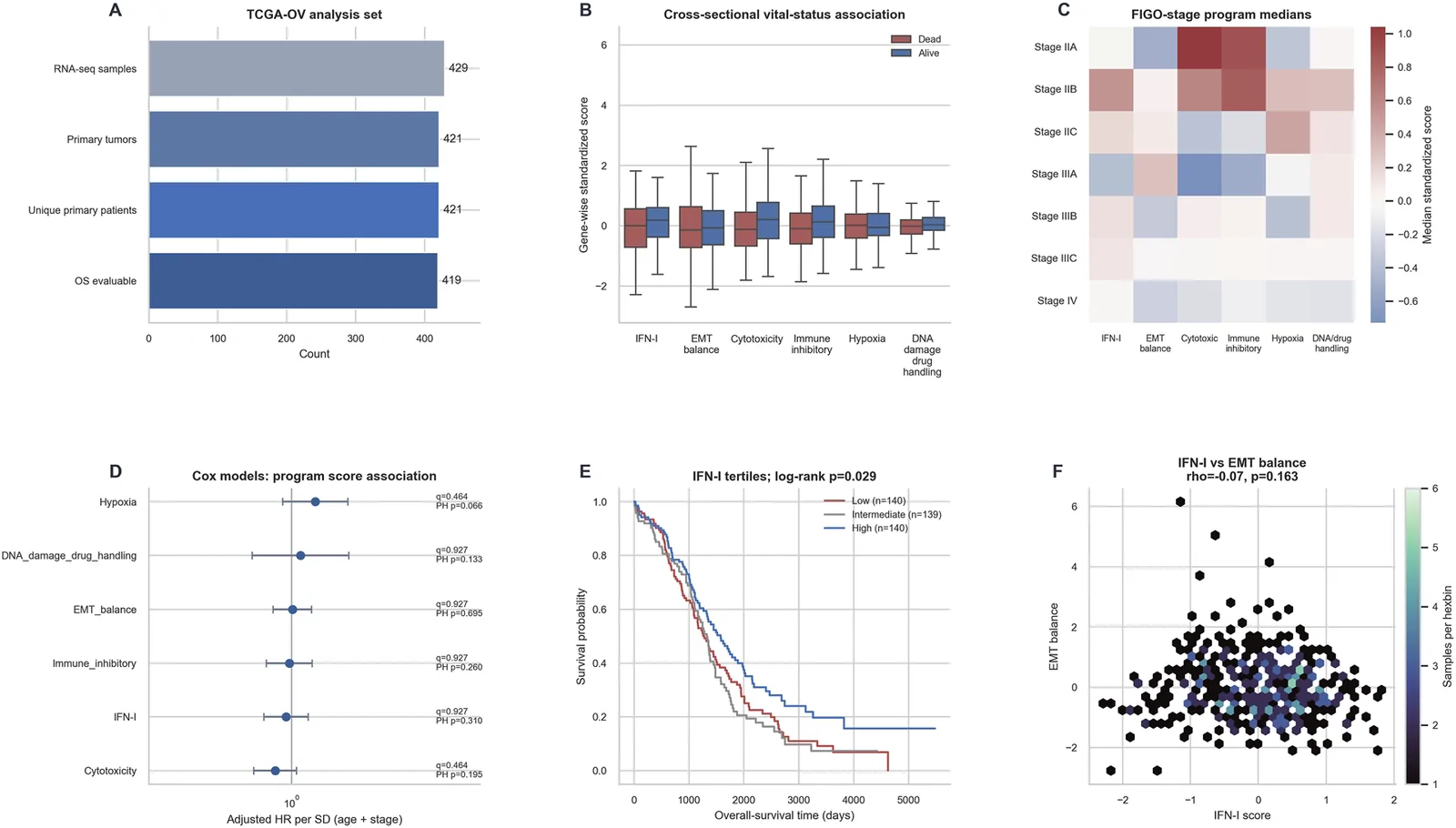
TCGA-OV transcriptomic and survival context. **(A)** Sample-selection flow. **(B)** Cross-sectional program distributions by vital status. **(C)** Median program scores across FIGO stages. **(D)** Age- and stage-adjusted Cox estimates per score; horizontal bars are 95% confidence intervals, and proportional hazard (PH) *p* values are from rank-based proportional-hazards tests. **(E)** Overall survival curves by IFN-I tertile. **(F)** Hexbin density of IFN-I and EMT-balance scores. Response-group comparisons are drawn from GSE300897.

### 3.8 Multiparametric MRI complements an interferon-high tumor transcriptome

The TCGA-24-1423 case links a multiparametric MRI examination to a TCGA-OV tumor RNA-seq sample. Pre-contrast T1 VIBE establishes baseline T1 signal (Figure 8A), while post-contrast and delayed T1 VIBE show vascular and solid-organ enhancement across successive phases (Figures 8B,C). Axial T2 HASTE emphasizes fluid-containing structures and soft-tissue contrast (Figure 8D). DWI and ADC provide complementary diffusion-weighted and apparent-diffusion-coefficient views of the same examination (Figures 8E, F). In the paired transcriptome, the IFN-I program was at the 86.8th TCGA-OV percentile, with IFIT1 at the 87.5th and MX1 at the 93.7th percentiles; hypoxia (52.9th) and EMT balance (46.4th) were near the cohort midpoint. The imaging series therefore provide a concrete multiparametric context for an interferon-high HGSC transcriptome.

**FIGURE 8:**
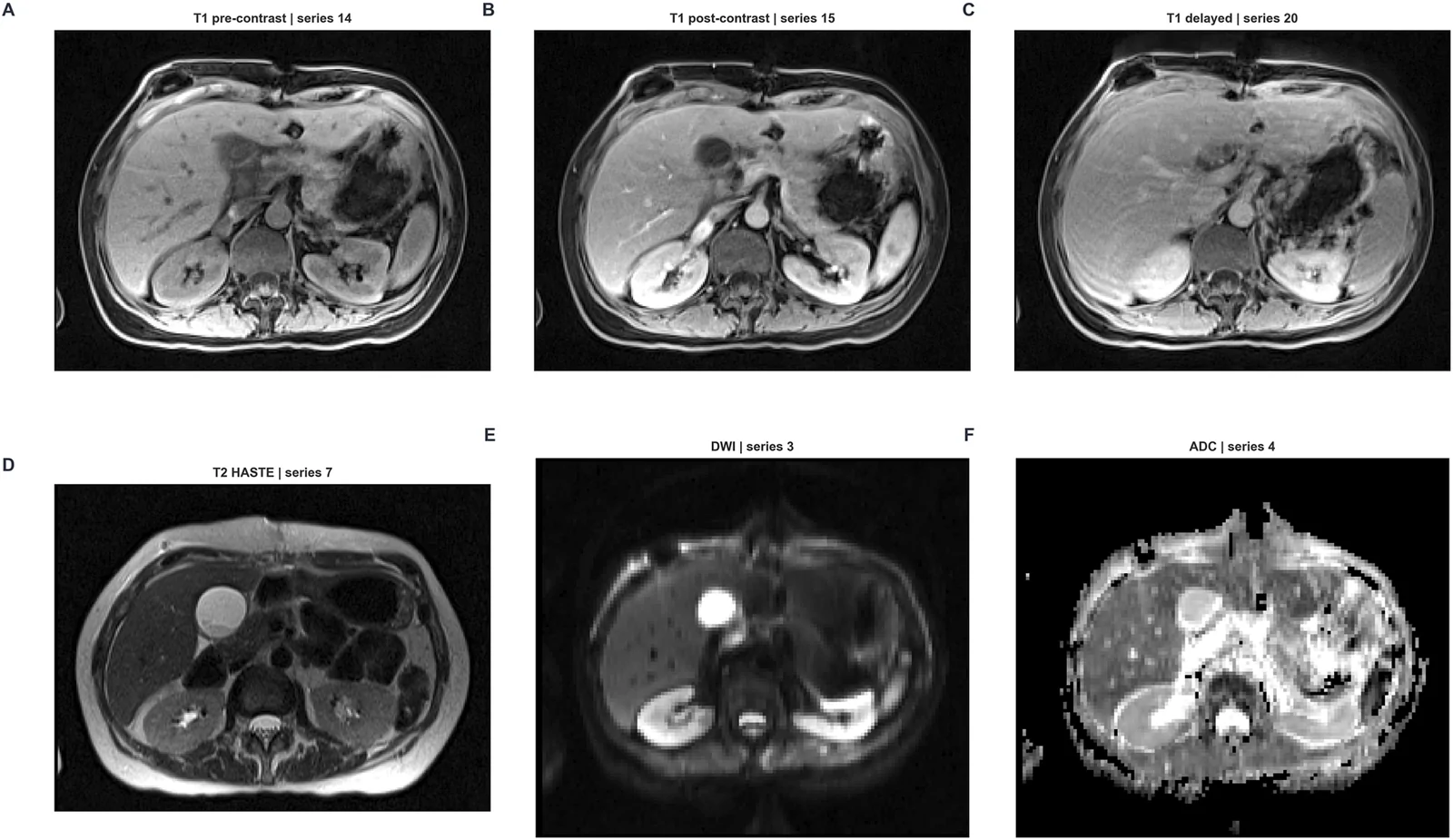
Barcode-matched multiparametric MRI and transcriptome profile for TCGA-24-1423. Representative central DICOM images show **(A)** T1 VIBE pre-contrast, **(B)** T1 VIBE post-contrast, **(C)** delayed T1 VIBE, **(D)** axial T2 HASTE, **(E)** DWI, and **(F)** ADC series. The contrast phases show vascular and parenchymal enhancement, T2 HASTE highlights fluid-sensitive soft-tissue contrast, and DWI/ADC add complementary diffusion information. The paired tumor transcriptome had an IFN-I score at the 86.8th TCGA-OV percentile, with IFIT1 and MX1 at the 87.5th and 93.7th percentiles. Figure was prepared from the TCGA-OV collection of The Cancer Imaging Archive (Holback et al., 2016).

### 3.9 Integrated interpretation

Across the nine GSE300897 libraries, no cancer-cell program passed BH *q* < 0.05; the largest directional shifts were lower IFN-I (−0.15) and lower EMT balance (−0.16) in refractory libraries, alongside a smaller increase in hypoxia (0.05). These program shifts occur within a visibly structured mixture of peritoneal, mesenteric, and omental biopsies. TCGA-OV adds cohort-scale expression and survival context, and the barcode-matched MRI case connects the transcriptomic analysis to real contrast-enhanced, T2-weighted, and diffusion acquisitions.

### 3.10 *In vitro* RT-qPCR results

Cisplatin-exposed OVCAR-3 cells showed lower relative IFIT1 (0.55 ± 0.03-fold; Holm-adjusted *p* = 0.00075) and MX1 (0.60 ± 0.04-fold; *p* = 0.00120), with higher SNAI1 (1.47 ± 0.06-fold; *p* = 0.00080) and HIF1A (1.64 ± 0.13-fold; *p* = 0.00120) compared with vehicle. Caov-3 cells showed the same directional pattern: IFIT1 0.68 ± 0.04-fold (*p* = 0.00337), MX1 0.68 ± 0.06-fold (*p* = 0.00693), SNAI1 1.41 ± 0.07-fold (*p* = 0.00337), and HIF1A 1.46 ± 0.13-fold (*p* = 0.00693). Values are mean ± SD from four independent cultures per condition. Thus, acute cisplatin exposure was associated with reduced IFIT1/MX1 expression and increased SNAI1/HIF1A expression in both ovarian cancer cell lines (Figure 9). These treatment-induced changes were analyzed as a separate perturbation experiment rather than as a direct refractory-versus-sensitive comparison.

**FIGURE 9:**
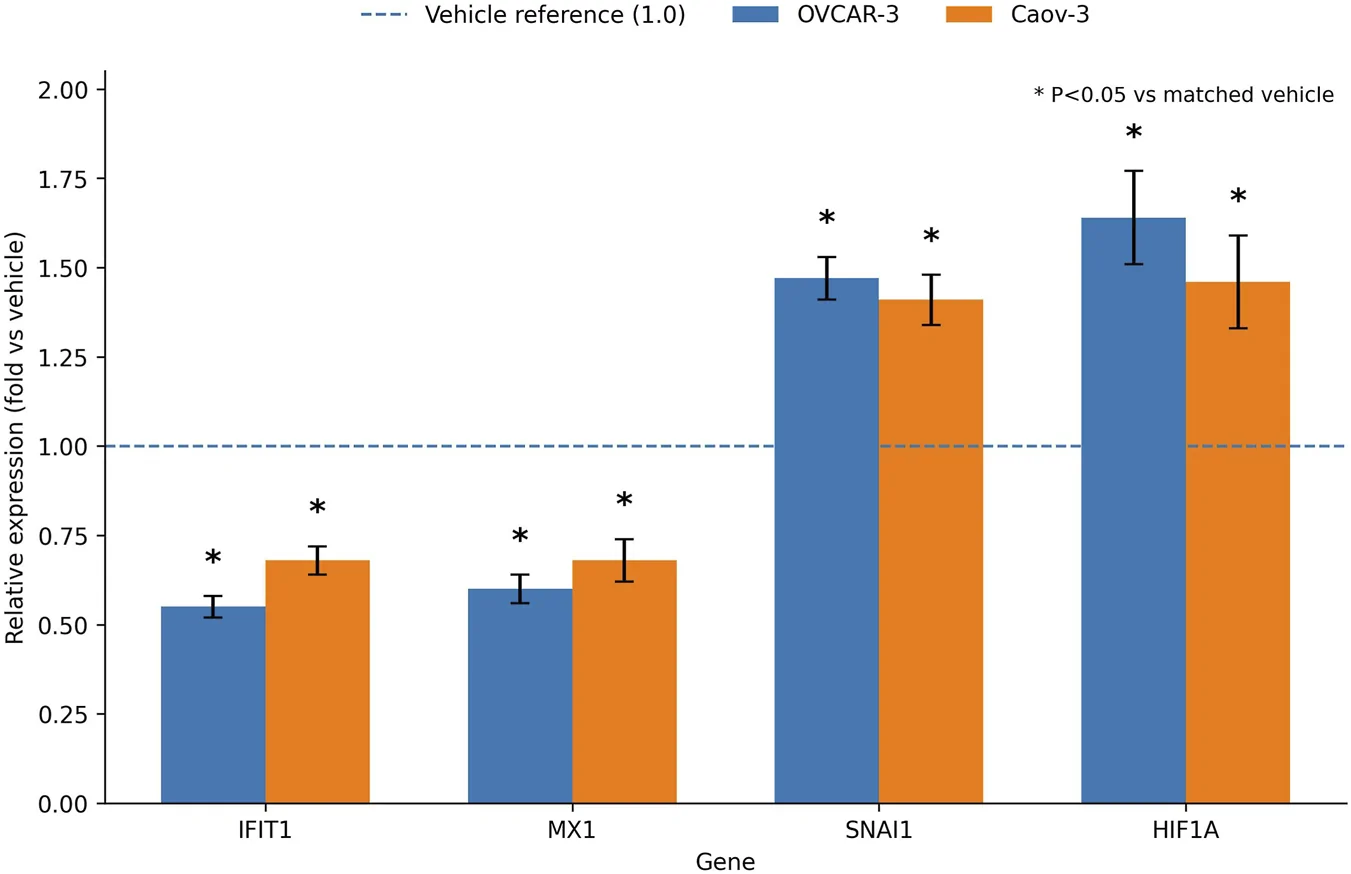
RT-qPCR analysis of cisplatin-induced expression changes in OVCAR-3 and Caov-3 cells. IFIT1, MX1, SNAI1, and HIF1A expression after cisplatin (5 μM, 24 h) was normalized to the vehicle condition (1.0) by the 2^−ΔΔCt^ method using GAPDH. Bars show the mean ± SD from four independent cultures per condition; each cDNA was analyzed in technical triplicate. Statistical significance was assessed on ΔΔC_t_ values using two-sided one-sample *t* tests against 0 with Holm adjustment across the four genes within each cell line; all displayed cisplatin-induced changes were significant at Holm-adjusted *p* < 0.05. The experiment represents an acute cisplatin perturbation and is not a direct refractory-versus-sensitive comparison.

## 4 Discussion

This reanalysis identifies several directional, but statistically non-definitive, patterns associated with chemotherapy refractoriness in the nine GSE300897 HGSC libraries. At the library/patient level, refractory cancer cells showed lower IFN-I scores and lower EMT balance, together with a small increase in hypoxia. Importantly, no cancer-cell program passed BH *q* < 0.05. The appropriate interpretation is therefore exploratory: the data prioritize biological axes for further study but do not establish a validated molecular classifier of refractory disease.

The direction of the IFN-I and hypoxia findings is broadly consistent with the source multi-omics study from which GSE300897 was generated, which reported attenuated IFN-I signaling and increased hypoxia-associated activity in chemo-refractory HGSC (Afenteva et al., 2025). This biological direction is also compatible with broader evidence linking tumor-cell IFN-I signaling to chemotherapy response and hypoxic adaptation to chemoresistance (Sistigu et al., 2014; Belisario et al., 2020). However, our simplified program score reanalysis is not an independent validation cohort. It uses the same public data source, a small number of patients, and a deliberately conservative library-level analytical unit. The absence of BH-significant program differences in the present analysis should therefore be reported alongside, rather than obscured by, the directional agreement with the source study.

The single-cell compartment analyses also emphasize that transcriptomic signals are composition-sensitive. T-cell, myeloid, fibroblast, malignant, and vascular populations carry distinct immune, hypoxia, extracellular matrix, and cytotoxic programs, consistent with marked cellular heterogeneity described across HGSC single-cell studies (Izar et al., 2020; Olbrecht et al., 2021; Xu et al., 2022; Qian et al., 2020). EMT-associated programs provide an additional descriptive axis but are context dependent and should not be treated as a single linear state (Nieto et al., 2016). These compartment-level patterns are useful for localization and hypothesis generation, but they do not by themselves establish intercellular causality or a treatment mechanism.

TCGA-OV provides a larger cohort-scale context but does not supply an independent chemotherapy response validation set for GSE300897. Among the 419 cases evaluable for the displayed survival analyses, age- and stage-adjusted Cox models were fitted in evaluable stage IIIC/IV cases; none of the six displayed program scores reached BH *q* < 0.05. Although IFN-I tertiles showed a nominal log-rank separation, continuous IFN-I was not significant after adjustment. Accordingly, the TCGA analyses should be regarded as descriptive survival context rather than evidence for a clinically validated prognostic or predictive biomarker. This conservative interpretation is consistent with the known genomic, proteomic, and subtype heterogeneity of HGSC (Cancer Genome Atlas Research Network, 2011; Tothill et al., 2008; Konecny et al., 2014; Chen et al., 2018; Zhang et al., 2016).

The TCGA-24-1423 example links real multiparametric MRI acquisitions to a matched tumor transcriptome and places that transcriptome within the TCGA-OV expression distribution. The relatively high IFN-I, IFIT1, and MX1 percentiles provide a concrete molecular context for the displayed examination. Nevertheless, one barcode-matched case cannot establish an MRI–transcriptome association, define an imaging phenotype of IFN-I activity, or support patient-level prediction. Prior multi-institutional ovarian radiogenomic work illustrates both the potential and the reproducibility challenges of deriving imaging–molecular relationships (Vargas et al., 2017), while MRI heterogeneity measurements themselves require standardized acquisition and analysis (Just, 2014; Holback et al., 2016).

The RT-qPCR experiment provided a separate *in vitro* perturbation test of selected genes related to the principal descriptive axes in this manuscript. OVCAR-3 and Caov-3 are established ovarian cancer cell models, although ovarian cancer cell lines vary substantially in their genomic resemblance to HGSC (Domcke et al., 2013; Beaufort et al., 2014). IFIT1 and MX1 were selected as markers of IFN-I activity, SNAI1 as an EMT-related marker, and HIF1A as a hypoxia-associated marker. Acute cisplatin exposure reduced IFIT1/MX1 and increased HIF1A, changes that are directionally compatible with the IFN-I and hypoxia axes highlighted in the public data analyses. SNAI1 also increased after cisplatin exposure, indicating an EMT-related treatment response; however, this single-gene change should not be interpreted as validation of the lower EMT-balance score observed in pretreatment refractory libraries. More broadly, the cell line experiment models an acute drug response, whereas GSE300897 compares pretreatment states associated with later clinical response. The RT-qPCR findings therefore provide orthogonal perturbation evidence for selected biological axes rather than independent validation of a patient-level predictive biomarker or proof of a causal resistance mechanism.

Several limitations define the inferential boundary of this study. First, GSE300897 contains only nine treatment-naïve libraries/patients, and chemotherapy status is imbalanced with anatomical sampling location; all three omental libraries belong to the sensitive group. Second, response comparisons are secondary analyses of public annotations and cannot remove unmeasured clinical or sampling confounding. Third, TCGA-OV lacks the same response definition and therefore provides contextual rather than direct validation evidence. Fourth, the MRI component is a single-case illustration rather than a radiogenomic cohort. Fifth, multiple program-level comparisons were performed, and the absence of BH-significant cancer-cell program differences should constrain biological claims. Sixth, the RT-qPCR experiment was limited to two established ovarian cancer cell lines, one cisplatin dose and exposure duration, and four independent cultures per condition. It measures acute treatment-induced expression changes rather than pretreatment clinical refractoriness. SNAI1 is also a single EMT-related marker and is not equivalent to the multigene EMT-balance score used in the transcriptomic analyses. The experiment therefore does not establish clinical predictive validity or a causal treatment-resistance mechanism. Larger independent HGSC cohorts with harmonized response definitions, balanced sampling, prospectively linked imaging and molecular data, and expanded orthogonal laboratory validation will be required for confirmation.

### 4.1 Implications and future directions

The present results nominate IFN-I, hypoxia, and related cell-state programs for further investigation in chemotherapy-refractory HGSC, but they do not justify treatment allocation or clinical risk scoring. Future work should test these programs in independent response-annotated single-cell cohorts, extend RT-qPCR and protein-level assays to additional genomically appropriate HGSC models and treatment conditions, incorporate perturbation experiments where mechanistic claims are intended, and assemble multi-patient MRI–transcriptome datasets with standardized imaging protocols. Such studies would permit formal assessment of reproducibility, incremental value, and any eventual predictive utility before clinical translation.

## 5 Conclusion

In this public data reanalysis, chemotherapy-refractory HGSC libraries showed directional reductions in cancer-cell IFN-I and EMT balance and a small increase in hypoxia, but no cancer-cell program passed BH correction. TCGA-OV provided cohort-scale transcriptomic and survival context but did not validate a robust program-level survival association. A single barcode-matched MRI case illustrated the feasibility of connecting multiparametric imaging with a tumor transcriptome but cannot establish an imagingbiomarker. *In vitro* RT-qPCR demonstrated lower IFIT1/MX1 and higher HIF1A/SNAI1 expression after acute cisplatin exposure in both OVCAR-3 and Caov-3 cells. The IFIT1/MX1 and HIF1A changes provide directionally compatible perturbation evidence for the IFN-I and hypoxia axes, whereas SNAI1 is interpreted as an EMT-related treatment response rather than validation of the lower pretreatment EMT-balance signal. The overall findings remain hypothesis-generating and support further validation in independent response-annotated and imaging-linked HGSC cohorts together with expanded laboratory experiments rather than immediate clinical application.

## Data Availability

The datasets presented in this study can be found in online repositories. The names of the repository/repositories and accession number(s) can be found in the article.
